# The health care and societal costs of inherited retinal diseases in Australia: a microsimulation modelling study

**DOI:** 10.5694/mja2.51997

**Published:** 2023-06-10

**Authors:** Deborah Schofield, Joshua Kraindler, Owen Tan, Rupendra N Shrestha, Sarah West, Natalie Hart, Liny Tan, Alan Ma, John R Grigg, Robyn V Jamieson

**Affiliations:** ^1^ GenIMPACT: Centre for Economic Impacts of Genomic Medicine, Macquarie Business School Macquarie University Sydney NSW; ^2^ Children's Medical Research Institute, Sydney Children's Hospitals Network University of Sydney Sydney NSW; ^3^ Save Sight Institute University of Sydney Sydney NSW; ^4^ The University of Sydney Sydney NSW

**Keywords:** Ophthalmology, Cost of illness, Retinal diseases

## Abstract

**Objectives:**

To estimate the health care and societal costs of inherited retinal diseases (IRDs) in Australia.

**Design, setting, participants:**

Microsimulation modelling study based on primary data — collected in interviews of people with IRDs who had ophthalmic or genetic consultations at the Children's Hospital at Westmead or the Save Sight Institute (both Sydney) during 1 January 2019 – 31 December 2020, and of their carers and spouses — and linked Medicare Benefits Schedule (MBS) and Pharmaceutical Benefits Schedule (PBS) data.

**Main outcome measures:**

Annual and lifetime costs for people with IRDs and for their carers and spouses, grouped by payer (Australian government, state governments, individuals, private health insurance) and type (health care costs; societal costs: social support, National Disability Insurance Scheme (NDIS), income and taxation, costs associated with caring for family members with IRDs); estimated annual national cost of IRDs.

**Results:**

Ninety‐four people (74 adults, 20 people under 18 years; 55 girls and women [59%]) and 30 carers completed study surveys (participation rate: adults, 66%; children, 66%; carers, 63%). Total estimated lifetime cost was $5.2 million per person with an IRD, of which 87% were societal and 13% health care costs. The three highest cost items were lost income for people with IRDs ($1.4 million), lost income for their carers and spouses ($1.1 million), and social spending by the Australian government (excluding NDIS expenses: $1.0 million). Annual costs were twice as high for people who were legally blind as for those with less impaired vision ($83 910 *v* $41 357 per person). The estimated total annual cost of IRDs in Australia was $781 million to $1.56 billion.

**Conclusion:**

As the societal costs associated with IRDs are much larger than the health care costs, both contributors should be considered when assessing the cost‐effectiveness of interventions for people with IRDs. The increasing loss of income across life reflects the impact of IRDs on employment and career opportunities.

**The known**: Inherited retinal diseases (IRDs) have significant cost implications, both for people with these disorders and for their families. Comprehensive information about the costs of IRDs in Australia, however, have not been published.

**The new**: Using a microsimulation model, we estimated an overall lifetime cost of $5.2 million per person with an IRD. Societal costs, including government support and lost income for people with impaired sight and their families, accounted for 87% of all costs, health care costs for only 13%.

**The implications**: To correctly assess the cost‐effectiveness of IRD treatments, including genomic testing and gene therapies, the substantial societal costs of IRDs must be taken into account.

Inherited retinal diseases (IRDs) comprise a group of heterogeneous monogenic disorders characterised by retinal dysfunction and degeneration, often leading to progressive vision loss and blindness.^1^ Genomic variant prevalence data suggest that IRDs affect about one in 1000 people,^2^, ^3^and IRDs are now the leading cause of blindness in working age adults.^4^ As early onset of disease often causes significant vision deficits during childhood and throughout life, their impact, both on those directly affected and on their families, is considerable.^5^

The limited treatment options in Australia have gradually improved since voretigene neparvovec (Luxturna, Novartis) was approved for people with *RPE65* retinopathy in 2020, the first publicly subsidised *in vivo* gene therapy in Australia;^6^ the first patient was treated in the same year.^7^ The Medical Services Advisory Committee (MSAC) acknowledged the clinical need for an effective treatment, but also noted some limitations, including in the cost–effectiveness analysis for the application (which did not take benefits for carers into account), and required a full review within three years of its approval.^8^

The impact of vision loss includes increased risk of poverty, reduced quality of life and employment opportunities, and greater expenses for informal care.^9^, ^10^, ^11^ Information on the total cost of IRDs, including use of health and social services, education, employment and income, is limited.^10^, ^12^ In the United States, mean annual health care costs for people with IRDs were US$7317 higher than for the general population,^13^ but this estimate, like those of other overseas studies,^14^, ^15^ did not take all health care (eg, allied health, vision aids) and other costs into account.

Two recent overseas investigations found that the annual national costs of IRDs are considerable: US$13.4–31.8 billion in the United States and CAN$1.6–6.7 billion in Canada,^16^ and £42.6 million in Ireland and £523.3 million in the United Kingdom.^17^ The costs associated with reduced workforce participation and quality of life were substantial, health care expenses comprising only 2–7% of total costs. However, these studies included people with non‐IRD vision impairment, included large loss of quality of life components in addition to disease costs, did not compare income losses for people with IRDs with mean population incomes, did not control for factors such as age and education, and relied on mean welfare payments and tax rates rather than collecting primary data on these items. Further, their online surveys of people with vision impairment were subject to selection biases (including vision capacity).

A health care system and societal perspective is needed when assessing the costs and benefits of new clinical interventions, including genomic testing and targeted therapies. However, no complete overview of the costs associated with IRDs in Australia has been reported. We therefore estimated the health and societal costs of IRDs, analysing both primary and administrative health care data for a group of people with IRDs and their carers. We used microsimulation modelling to estimate income losses, important flow‐on costs (eg, subsidies for housing), and National Disability Insurance Scheme (NDIS) costs.

## Methods

Our microsimulation modelling study is one component of the Economic and Psychosocial Impact of Caring for Families Affected by Visual Impairment (EPIC‐Vision) study. All people with clinical IRD diagnoses who had ophthalmic or genetic consultations at the Children's Hospital at Westmead or the Save Sight Institute (Sydney Eye Hospital campus, University of Sydney) during 1 January 2019 – 31 December 2020 and their carers or partners were invited to participate. Specialist clinicians interviewed participants using the EPIC‐Vision questionnaire, a tailored IRD questionnaire designed in consultation with ophthalmologists, clinical geneticists, genetic counsellors, and health economists; it includes questions on social services use (government support, NDIS) and other costs (education, aids, and modifications). When appropriate, carers were also interviewed on behalf of people with IRDs, including all those under 18 years of age.

### Costs analysis

Costs data for our simulation model were derived from the EPIC‐Vision questionnaire responses and from linked Medicare Benefits Schedule (MBS) and Pharmaceutical Benefits Schedule (PBS) data. This information was provided to Services Australia, which performed the data linkage and supplied de‐identified data to the investigators. The health care‐related, income and employment, and societal parameters included and their data sources, together with the indices used for adjustment to December 2022 prices, are summarised in the Supporting Information. All analyses were undertaken in SAS 9.4.

### Income and tax effects

We estimated the income and tax effects of IRDs in a static income model (STINMOD) based on a representative sample of people and households in Australia (Box 1). People with IRDs and their carers and partners were matched in STINMOD with respect to age, gender, and highest level of education, and 1000 simulations run for each person. The mean outcomes were then used as the counterfactuals for each participant (ie, the estimated value if they did not have an IRD), and the effect of the IRD defined as the difference between the simulation outcomes and EPIC‐Vision data for the participant. STINMOD data reflect income and tax levels at the end of 2019.^18^

Box 1 **The microsimulation model used for estimating income and tax costs associated with inherited retinal diseases**

**Figure:**
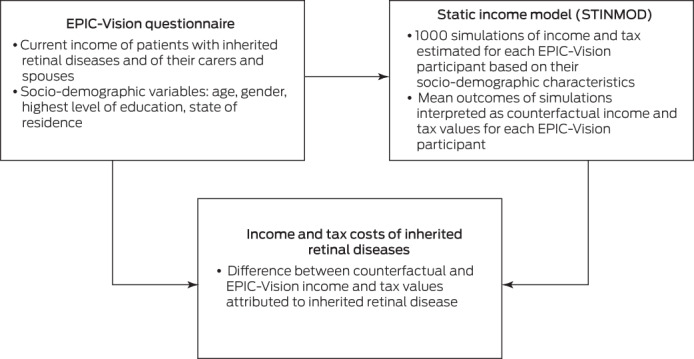
EPIC‐Vision = Economic and Psychosocial Impact of Caring for Families Affected by Visual Impairment study.

### Household and lifetime cost

Participants were grouped by age (0–5, 6–18, 19–29, 30–39, 40–49, 50–59, 60–69, 70–80 years). Total lifetime costs were calculated from the mean costs for each age group. As none of the participants were over 80 years of age, we used mean annual costs for the 70–80 year age group to project costs to the mean life expectancy age in Australia (84 years).^19^ The estimated total costs for each person diverge from the sum of the component costs because of an adjustment that reflects the fact that as income declines for an individual, they receive increasing welfare support.

### Visual acuity and inherited retinal disease diagnosis

To assess whether costs differed by visual acuity, we estimated a model in which people were grouped as to whether they were eligible to be registered as being legally blind in Australia (Snellen visual acuity poorer than 6/60) or had measured visual acuity of better than normal to moderate vision impairment (6/60 or better).

### Health and societal costs

Health costs are costs related directly to the health system and use of health products such as pharmaceuticals. Societal costs refer to all other costs, including income losses, as well as government service and NDIS costs. We report the proportions of all costs attributed to health and societal costs.

### Total national costs

Because of uncertainty regarding the prevalence of IRDs, we used two estimated values for estimating total national costs: 0.1%^1^ and 0.05%.^20^

### Ethics approval

The EPIC‐Vision study was approved by the Sydney Children's Hospitals Network Human Research Ethics Committee (HREC/18/SCHN/292).

## Results

Of 112 adults invited to participate in the study, 74 agreed to do so (66%) and 71 completed the survey (63%). Their mean age was 42.1 years (standard deviation [SD], 17.3 years) and 47 were women (66%). The visual acuity of 39 participants (53%) was less than 6/60 (legally blind). Twenty‐two people were working full‐time (31%), 22 part‐time (31%), eight were unemployed and looking for work (11%), and nineteen were unemployed and not looking for paid work (27%); for eighteen of those not in paid employment, health was cited as the reason. Four participants were Aboriginal or Torres Strait Islander people (6%; 2021 census: 3%^21^) and nineteen were not born in Australia (28%; 2021 census: 29%^22^); 29 had NDIS plans (41%) (Box 2).

Box 2 **Characteristics of the people with inherited retinal disease and their carers who participated in the EPIC‐Vision survey, 2019 and 2020**

**Table:** 

	People with inherited retinal diseases		
Characteristic	Adults*	Children^†^	Carers
Participants	74	20	30
Age (years), mean (SD)	42.1 (17.3)	11.0 (4.6)	47.0 (11.0)
Gender			
Women	47 (64%)	8 (40%)	25 (83%)
Men	27 (36%)	12 (60%)	5 (5%)
Aboriginal or Torres Strait Islander Australians^‡^	7 (6%)	NA	0
Born in Australia^‡^			
Yes	50 (70%)	NA	16 (53%)
No	19 (27%)	NA	14 (47%)
No response	2	NA	0
Visual acuity			
> 6/18 (normal to mild visual impairment)	15 (20%)	5 (25%)	NA
6/18 to 6/60 (moderate visual impairment)	20 (27%)	5 (25%)	NA
< 6/60 (legally blind; severe visual impairment)	22 (30%)	< 5	NA
< 3/60 (legally blind; profound visual impairment)	17 (23%)	6 (30%)	NA
Inherited retinal disease diagnosis^§^			
Rod‐dominated inherited retinal disease	34 (46%)	< 5	NA
Macular dystrophy	15 (20%)	< 5	NA
Cone‐dominated inherited retinal disease	12 (16%)	5 (25%)	NA
Inherited retinal disease, and a systemic disorder	13 (18%)	9 (45%)	NA
National Disability Insurance Scheme plan			
No, awaiting decision; or applied and did not receive a package	7 (9%)	< 5	NA
No, I have never applied	35 (47%)	6 (30%)	NA
Yes	32 (43%)	12 (60%)	NA
Employment status^‡^			
Full‐time paid work	22 (31%)	NA	10 (33%)
Part‐time paid work	22 (31%)	NA	9 (30%)
Unemployed, looking for paid work	8 (11%)	NA	< 5
Unemployed, not looking for paid work	19 (27%)	NA	8 (27%)
Reason for being out of work^‡^			
Ill health or disability	18/27 (67%)	NA	NA
Caring for child/relative with inherited retinal disease	NA	NA	5 (50%)
Other	9/27 (33%)	NA	5 (50%)
Main source of income^‡^			
Wage/salary	33 (46%)	NA	NA
Self‐employed	5 (7%)	NA	NA
Government benefits	25 (35%)	NA	NA
Investments/superannuation	5 (7%)	NA	NA
Other or no income	< 5	NA	NA
Highest education level^‡^			
Less than year 12	8 (11%)	NA	5 (17%)
Year 12	14 (20%)	NA	0
Trade/apprenticeship	5 (7%)	NA	0
Certificate/diploma	12 (17%)	NA	6 (20%)
University degree	20 (28%)	NA	12 (40%)
Higher university degree	12 (17%)	NA	7 (23%)

EPIC‐Vision = Economic and Psychosocial Impact of Caring for Families Affected by Visual Impairment; NA = not applicable; SD = standard deviation.

* Includes three adults for whom carers completed the survey as proxies; for the characteristics marked with a double dagger (see below), the denominator for proportions is consequently 71, not 74. For seven cases in which both the person with an IRD and their carer completed the survey question on costs, the responses of the person with an IRD were used.

† Completed by their family carers as proxies.

‡ Asked only of adults completing the survey on their own behalf.

§ Rod‐dominated inherited retinal disease includes rod–cone dystrophy, retinitis pigmentosa, choroideraemia, enhanced S‐cone syndrome, and Leber congenital amaurosis. Macular dystrophy includes Stargardt diseases and other macular dystrophy types. Cone‐dominated IRD includes cone‐rod dystrophy, cone dystrophy, and achromatopsia. IRD plus systemic disorder includes Usher syndrome and other.

Carers completed surveys on behalf of ten adults with IRDs; the mean age of these adults was 31.7 years (SD, 17.6 years), and visual acuity less than 6/60 vision for nine. Seven of these adults also completed surveys themselves; their responses, not the proxy responses of their carers, were included in our analysis.

Of thirty people under 18 years invited to participate, the carers of twenty agreed on their behalf and completed the surveys (66%). Their mean age was 11.0 years (SD, 4.6 years) and eight were girls (40%). The visual acuity of ten children (50%) was less than 6/60 (legally blind); twelve had NDIS plans (60%) (Box 2).

Of 47 carers invited to participate in the study, thirty agreed to do so and completed the surveys (63%). Their mean age was 47.0 years (SD, 11.0 years) and 25 were women (83%). Nineteen carers had university degrees (63%); ten had full‐time employment (33%), nine had part‐time work (30%), and eleven (30%) were not employed (Box 2).

### Overall costs

The total estimated lifetime cost was $5.2 million per person with an IRD and $5.6 million per household. The mean annual cost was highest for the 50–59‐year age group ($96 167), attributable to income loss for both people with IRDs and for their spouses and carers. The mean annual cost was lowest for the 18–29 (smaller income losses) and 70–80‐year age groups (not eligible for NDIS plans) (Box 3). Of total costs, 87% were societal costs and 13% health care costs. The three highest cost items were $1.4 million in lost income for people with IRDs, $1.1 million in lost income for their carers and spouses, and $1.0 million in social spending by the Australian government (excluding NDIS expenses) (Box 4). The mean annual costs were about 40% higher for male than female participants (Box 5), including greater loss of income and NDIS expenses (data not shown).

Box 3 **Estimated mean annual costs associated with inherited retinal diseases (IRDs), per person, by age group and expense type (Australian dollars, December 2022 prices)**

**Table:** 

	Age group (years)							
Characteristic	0–5	6–18	19–29	30–39	40–49	50–59	60–69	70–84
People with IRDs	6	17	21	9	15	14	6	6
**Costs for person with IRD**								
Australian government								
Health system	15 859	1675	874	9767	2003	2693	3382	2 522
Social support	1335	7955	11 430	10 889	10 999	15 653	16 683	17 025
National Disability Insurance Scheme	7948	7844	4106	7895	5365	3910	7612	—
Tax receipts	—	282	599	9015	11 535	17 198	8373	333
State government								
Health system	14 482	272	158	9020	933	1000	1079	615
Social support	4004	4314	—	—	—	—	333	—
National Disability Insurance Scheme	5755	5680	2973	5717	3885	2831	5512	—
People with IRDs								
Health care costs	3655	1198	894	825	1968	1533	2108	912
Other	3266	2646	544	343	416	793	1210	1137
Lost income	—	1263	4286	31 163	29 009	44 494	29 028	2853
Other (private hospital services)	79	56	45	105	221	1181	1339	630
**Costs for carers and spouses**								
Australian government								
Social support	9356	13 721	2348	751	—	—	563	—
Tax receipts	4587	11 122	3889	200	577	5250	4043	2074
Carers and spouses								
Lost income	16 210	32 408	11 796	1112	1185	14 841	15 862	12 518
**Total annual costs** *	77 181	74 185	34 798	75 215	57 320	96 167	80 650	23 788
**Lifetime costs** ^†^								
Per person	385 906	1 350 313	1 733 090	2 485 243	3 058 440	4 020 110	4 826 609	5 159 644
Per household	416 372	1 456 916	1 869 913	2 681 447	3 299 896	4 337 487	5 207 657	5 566 985

* The estimated total costs will diverge from the sum of the components because of adjustment to reflect the fact that as income declines for an individual, they receive welfare support.

† To the end of the corresponding age bracket. The household value assumes that each household statistically includes slightly more than one person with an inherited retinal disease.

Box 4 **Estimated lifetime costs associated with inherited retinal diseases, per person, by expense type and payer (Australian dollars, December 2022 prices)**

**Table:** 

Characteristic	Value
Australian government	2 855 820
Health care	324 429
Social support* (person)	1 016 419
National Disability Insurance Scheme	434 688
Social spending (carer)	264 113
Tax receipts (person)	476 140
Tax receipts (carer/spouse)	340 031
State government	600 828
Health care	206 614
Social support (person)	79 439
National Disability Insurance Scheme	314 774
Person/household	2 798 860
Health care^†^	120 789
Other, including aids/modifications	100 254
Lost income (person)	1 440 453
Lost income (spouse/carer)	1 137 364
Private health insurance costs^‡^	38 893
Total cost (after adjustment)^§^	5 159 644

* Excludes National Disability Insurance Scheme expenses.

† Medicare co‐payments, orthoptics, alternative medicines, transport and accommodation costs linked with hospital stays.

‡ For hospital services.

§ The estimated total costs will diverge from the sum of its components because of adjustment to reflect the fact that as income declines for an individual, they receive welfare support.

Box 5 **Estimated annual costs for people with inherited retinal diseases, per person, by gender and visual acuity (Australian dollars, December 2022 prices)**

**Table:** 

Characteristic	Value
Gender	
Girls/women (*N* = 55)	54 527
Boys/men (*N* = 39)	76 067
Visual acuity	
Better than or equal to 6/60 (*N* = 45)	41 357
Less than 6/60 (*N* = 49)	83 910

### Health care costs

Total lifetime health care costs were estimated to be $690 725 per person with an IRD: $324 429 borne by the Australian government (47%), $206 614 by state governments (30%), $120 789 by people with IRDs (17%), and $38 893 by private insurance (6%) (Box 4). Lifetime health care costs were highest for the 0–5 year age group (higher health service use) and the 30–39 year age group (explained by extended hospital stays for one person with an IRD) (Box 3).

### Income and taxes

Total estimated lifetime loss of income was $2.6 million per person with an IRD (people with IRDs, $1 440 453 [56%]; carers and spouses, $1 137 364 [44%]) (Box 4). Mean annual lost income for people with IRDs aged 19–30 years was $4286, and peaked at $44 494 for those aged 50–59 years (Box 3). Total estimated lifetime loss in federal tax receipts was $816 171 (Box 4); the highest mean annual loss was for people aged 50–59 years (Box 3).

### Federal government social and welfare support

Total estimated lifetime Australian government social support costs (excluding the NDIS) was $1.0 million per person with an IRD; total lifetime NDIS costs for a person with an IRD were $749 462 (Box 4). Social support costs were generally higher for older age groups (Box 3). The estimated lifetime cost to the federal government for social support for carers and spouses was $264 113 per person with an IRD (Box 4), primarily associated with caring for a person under 18 years of age with an IRD (Box 3).

### Visual acuity

The total estimated annual cost for people with visual acuity poorer than 6/60 (ie, legally blind) were $83 910 per person, more than twice those for people with IRDs but whose visual acuity was at least 6/60 (Box 5); the difference was attributable to greater lost income and higher social support costs (including NDIS costs). However, total health care costs were slightly lower for the people with visual acuity poorer than 6/60 (data not shown).

### Total national annual cost

The estimated total annual cost attributable to IRDs in Australia was $781 million to $1.56 billion. After adjusting for the larger proportion of female participants with IRDs in our study, the estimated annual cost was $830 million to $1.66 billion (data not shown).

## Discussion

On the basis of primary and linked administrative data, we estimate that the lifetime cost associated with an IRD is $5.2 million per person, or a total of $781 million to $1.56 billion per year. Of these costs, 87% are societal costs and 13% health care costs.

Previous studies have found that vision loss in general is associated with substantial costs.^11^, ^12^, ^23^, ^24^ Interpreting the findings of two recent studies specifically concerned with IRDs^16^, ^17^ is made difficult by their data limitations and imprecise estimates of income losses. In our study, microsimulation modelling overcame these limitations by matching a group of people with diagnosed IRDs and their carers with people in the general population with similar characteristics rather than relying on comparisons with mean population income. This approach provides more precise estimates of the income and tax values we would expect for our participants if they did not have IRDs. Further, we found that estimated costs were highest for the 50–59‐year age group, primarily because of lost income.

People with IRDs receive a mixture of NDIS services, and their lifetime NDIS costs are greater than their health care costs. As people with NDIS plans before the age of 65 years can choose to remain in the NDIS beyond this age, our modelling would underestimate costs for people over 65 were the NDIS to support more services in the future than are currently available to people over 65 years of age.

We found that IRDs are associated with substantial income losses, exceeding $44 000 per year for people aged 50–59 years. This finding was consistent with comments by survey participants that they worked less because of their IRD or had left the workforce entirely. This suggests that helping people with IRDs with employment could improve societal outcomes. The impacts on both carers and spouses were also notable. For carers, lost income was largely linked with caring for children with IRDs. Although spouses might not be full‐time carers, the burden could nevertheless affect their ability to undertake paid work.

Health care costs were slightly higher for people with better vision, but overall costs were much higher for those who were legally blind. Societal costs must be considered when assessing the costs and benefits of IRDs, as health care cost estimates may not capture all costs.

Out‐of‐pocket health expenses comprised a small portion of total health costs, consistent with the lack of treatment options available for people with IRDs. They also spend substantial amounts on housing and transport modifications, and they may not be aware of all support provided by health care, the NDIS, and other agencies and consequently not receive appropriate support. Further, as some data were collected during the early phases of the coronavirus disease 2019 pandemic, our estimates of service use and related costs may be conservative.

### Limitations

The data for our study were collected from a large group of people with IRDs, allowing a comprehensive assessment of costs. Further, our simulation model was designed to be adaptable and applicable to other medical conditions. However, as the proportion of women in our study was larger than in the Australian population, income loss may have been underestimated, given the higher employment and income rates of men and the greater use of health services by women. Finally, we did not include any people near the end of life living in institutions such as nursing homes. The health care and other costs for people with IRDs near the end of life require further investigation.

### Conclusion

In our simulation study, we identified that IRDs are associated with substantial health care and societal costs. Given that 87% of the overall costs were societal, largely related to lower rates of employment for both patients and carers and their greater need for social support, it is crucial that societal costs are considered by cost‐effectiveness evaluations of future IRD interventions, including genomic testing and targeted therapies.

## Open access

Open access publishing facilitated by Macquarie University, as part of the Wiley – Macquarie University agreement via the Council of Australian University Librarians.

## Competing interests

Deborah Schofield, Robyn Jamieson, and John Grigg are supported by grant funding from the NHMRC (APP1116360) and the Luminesce Alliance. John Grigg and Robyn Jamieson have received consultancy fees from Novartis and are principal investigators in clinical trials undertaken by Belite Bio and Nacuity Pharmaceuticals. Robyn Jamieson is named on Australian provisional patent applications 2020904689 (AAV capsids and vectors) and 2022903363 (Treatment methods for inherited retinal diseases).

## Supporting information


Supplementary methods and results
