# Supplementary material for: The health care and societal costs of inherited retinal diseases in Australia: a microsimulation modelling study
Source: Med J Aust. 2023 Jun 10;219(2):70–6. doi: 10.5694/mja2.51997 (PMC10952471; doi:10.5694/mja2.51997)
Supplement: Supplementary file 1 — Supplementary methods and results [file MJA2-219-70-s001.pdf]

The Medical Journal of Australia

Supporting Information

Supplementary methods and results

This appendix was part of the submitted manuscript and has been peer reviewed.
It is posted as supplied by the authors.

Appendix to: Schofield D, Kraindler J, Tan O, et al. The health care and societal costs of inherited retinal diseases in Australia: a microsimulation modelling study. *Med J Aust* 2023; doi: 10.5694/mja2.51997.

Table. Data sources and methods for societal and health costs of inherited retinal diseases

Items	Cost and source	Perspective
**Health care**		
Out-of-hospital Medicare-subsidised services	MBS item number, schedule fee, and out-of-pocket cost. Data received are from year of interview and preceding four years; annual mean calculated for model.<br>EPIC-Vision survey*	Federal government; individual
Prescription medicines	PBS item number and out-of-pocket cost<br>Data received are from year of interview and preceding four years; annual mean calculated for model.<br>EPIC-Vision survey*	Federal government; individual
Non-prescription medicines and supplements	EPIC-Vision survey	Individual
Public inpatient care	Hospital use, mean cost of NSW bed-day	Federal and state governments
Private inpatient care	Hospital use, mean bed-day cost (private hospital), MBS item number	Federal government; private medical insurance; individual
Emergency department care	Emergency attendance, mean emergency attendance cost in NSW	Federal and state governments
Transport, accommodation and food during hospital care	EPIC-Vision survey	Individual
Allied and other health services (excluding MBS); eg, orthoptics	EPIC-Vision survey; imputed if use was reported without costs	Federal government; individual
**Income and employment**		
Employment status	EPIC-Vision survey	Individual
Income loss	EPIC-Vision survey; STINMOD	Individual
Tax receipts	EPIC-Vision survey; STINMOD	Federal government
**Societal costs**		
National Disability Insurance Scheme (NDIS) and social support		
Specialist disability accommodation	NDIS price guide[1]	Federal government
Supported independent living	EPIC-Vision survey; NDIS price guide	Federal government
Respite care	EPIC-Vision survey; NDIS price guide	Federal government
Rental support	EPIC-Vision survey; current rent subsidies[2]	Federal government
Public housing	EPIC-Vision survey;	State governments
NDIS package†	EPIC-Vision survey; – Current NDIS package amount	Federal and state governments, split according to 2021–22 federal budget[3]
Income and welfare support		
Income support, including Jobkeeper allowance, aged and disability pensions	EPIC-Vision survey; Department of Social Services Social Security Guide[2]	Federal government
Primary and secondary school support	EPIC-Vision survey; Department of Education, Skills and Education funding guides[4]	Federal and state governments
Early intervention class	EPIC-Vision survey; NDIS price guide	Federal and state governments
Preschool	EPIC-Vision survey; Department of Education, Skills and Education funding guides[4]	Federal and state governments

Items	Cost and source	Perspective
Aids and modifications		
Household aids and modifications	EPIC-Vision survey	Federal government; individual
Education aids and modifications	EPIC-Vision survey	Federal government; individual
Transport aids and modifications	EPIC-Vision survey	Federal government; individual
Guide dogs	EPIC-Vision survey; cost of training[5]	Federal government; individual

EPIC-Vision = Economic and Psychosocial Impact of Caring for Families Affected by Visual Impairment study; MBS = Medicare Benefits Schedule; NDIA = National Disability Insurance Agency; PBS = Pharmaceutical Benefits Scheme; STINMOD = Static income model.

Wages are adjusted for inflation using the Wage Prices Index.[6] Health costs are adjusted using annual health inflation from the Australian Institute of Health and Welfare (AIHW),[7] as well as index factors for the Medicare Benefits Schedule.[8] NDIS costs are adjusted using NDIS inflation.[9] Other costs are adjusted using the Consumer Price Index (CPI).[10]

* For people who did not consent to data linkage, estimated costs were based on their responses to the EPIC-Vision survey, in which participants were asked about their use of all types of health services, including Medicare-subsidised and non-subsidised services.

† Participants were asked about total NDIS package and individual NDIS spending amounts. If NDIS costs were specifically estimated elsewhere and included as part of other government spending, they were deducted from NDIS package amounts to avoid double counting.

References

1 National Disability Insurance Scheme. Pricing arrangements and price limits 2022-23. Canberra: NDIS 2022. https://www.ndis.gov.au/media/5917/download?attachment (viewed Feb 2023).

2 Services Australia. A guide to Australian government payments, 4 November 22 to 31 December 2022. Canberra: Services Australia 2022. https://www.servicesaustralia.gov.au/sites/default/files/2022-11/co029-2211.pdf (viewed Feb 2023).

3 Hewett R, King S. Funding the National Disability Insurance Scheme. Parliamentary library briefing book: key issues for the 47th Parliament. 2022. Canberra, Parliamentary library. https://www.aph.gov.au/About_Parliament/Parliamentary_departments/Parliamentary_Library/pubs/BriefingBook47p/FundingNDIS (viewed Nov 2022).

4 Department of Education, Skills and Employment. What is the Australian government doing to support students with disabilities in schools? Canberra: DESE, 2022. https://www.education.gov.au/download/3778/what-australian-government-doing-support-students-disability-schools/30121/what-australian-government-doing-support-students-disability-schools/pdf (viewed Nov 2022)

5 Guide Dogs NSW/ACT [website]. About our Guide Dogs. Sydney: Guide Dogs NSW/ACT. https://nsw.guidedogs.com.au/our-dogs/guide-dogs (viewed Nov 2022).

6 Australian Bureau of Statistics. Wage price index, Australia. Canberra: ABS, 2022. (ABS Cat. No. 6345.0). https://www.abs.gov.au/statistics/economy/price-indexes-and-inflation/wage-price-index-australia/latest-release (viewed Mar 2023).

7 Australian Institute of Health and Welfare. Health expenditure Australia 2020-21 Canberra: AIHW, 2022. (AIHW Cat. No. HWE 89). https://www.aihw.gov.au/reports/health-welfare-expenditure/health-expenditure-australia (viewed Feb 2023).

8 Department of Health and Aged care. MBS Online, Medicare Benefits Schedule. July 2022 News. Canberra: Department of Health and Aged Care 2022. http://www.mbsonline.gov.au/internet/mbsonline/publishing.nsf/Content/news-220701 (viewed Jan 2023).

9 National Disability Insurance Scheme. Annual financial sustainability report 2021-22. Canberra: NDIS 2022. https://www.ndis.gov.au/media/5567/download?attachment (accessed Feb 2023).

10 Australian Bureau of Statistics. Consumer price index, Australia. Canberra: ABS; Dec-quarter-2022. (ABS Cat. No. 6401.0). https://www.abs.gov.au/statistics/economy/price-indexes-and-inflation/consumer-price-index-australia/latest-release (viewed Feb 2023).
